# Decoding motor imagery EEG signals by a deep learning network integrating multiscale filtering and temporal-channel attention mechanisms

**DOI:** 10.1371/journal.pone.0361026

**Published:** 2026-10-23

**Authors:** Yong Zhu, Yongge Shi, Jianhua Wu, Qingguo Wei

**Affiliations:** 1 Department of Information Engineering, Gongqing College of Nanchang University, Jiujiang, China; 2 School of Information Engineering, Jiujiang Polytechnic University of Science and Technology, Jiujiang, China; 3 School of Information Engineering, Nanchang University, Nanchang, China; Nanyang Technological University, SINGAPORE

## Abstract

Due to their low signal-to-noise ratio and non-stationarity, accurately classifying EEG signals is still one of the main hindrances that limit the real-world applications of a brain-computer interface (BCI). Deep learning (DL) is a potential method for addressing the problem. However, how to create a suitable DL network for motor imagery (MI)-based BCIs poses severe challenges in the case of insufficient training data. In this paper, a DL network based on multiscale convolutional neural network (CNN) and temporal and channel attention mechanisms is proposed for decoding MI tasks. Temporal and spatial features are extracted by four parallel CNN branches, each of which is followed by a Transformer block for learning the global correlations of local time features. The outputs from the four Transformer blocks are concatenated along channel dimension and then input into a channel attention module for channel fusion. The subsequent feature map is classified by a CNN, which replaces the commonly used fully connected layer for reducing computational complexity. The proposed network was evaluated on three publicly available BCI datasets and compared with five state-of-the-art DL networks. The training data sets are augmented using the approach segmentation and reconstruction. The experimental results demonstrate that the proposed network achieved an average accuracy of 80.21% on Dataset 1 (4-class tasks), 90.19% on Dataset 2 (2-class tasks), and 97.32% on Dataset 3 (4-class tasks), surpassing the strongest baselines FBCNet on Dataset 1, MSFBCNN on Dataset 2 and MSFBCNN on Dataset 3 by 2.59%, 1.27%, and 1.99% respectively. The network effectively decoded the MI-based EEG signals, providing a potential method for BCI applications.

## 1. Introduction

A brain-computer interface (BCI) is an intelligent system that enables direct communication between the brain and a computer or other external devices [1]. The system collects brain electrical signals, decodes these signals and converts them into the control commands understandable by computers. In the past few decades, BCIs have developed rapidly, and BCI-related technologies have been widely applied in medical, entertainment, neural rehabilitation, and other fields [2–5]. Since electroencephalogram (EEG) has the advantages of noninvasiveness, cost efficiency and high time resolution, EEG-based BCIs have become one of the most vibrant areas of research.

Motor Imagery (MI) is the mental rehearsal of movement execution without any physical movement or the need of external stimuli. Thereby, an MI-based BCI (MI-BCI) is one kind of most intuitive and natural BCI paradigm. When a subject performs an MI task such as imagining movement of left hand or right hand, the contralateral primary sensorimotor cortex exhibits a decrease in amplitude of the μ (8–13 Hz) and β (14–30 Hz) rhythms, referred to as event-related desynchronization (ERD) [6,7]. The ipsilateral primary sensorimotor cortex, on the other hand, shows an increase in amplitude of the same rhythms, termed event-related synchronization (ERS) [8,9]. This differentiation in rhythmic amplitude can be leveraged to distinguish different MI tasks and thus constitutes the basis of creating an MI-BCI system. Since EEG signals are nonstationary and possess low signal-to-noise ratio, one of main challenges faced by MI-BCIs in real-world applications is to accurately recognize different MI tasks.

EEG signal decoding can be performed by either traditional machine learning (ML) or deep learning (DL) methods. As a typical pattern recognition system, a BCI includes two main signal processing steps, namely feature extraction and pattern classification. The traditional ML algorithms for feature extraction include Common Spatial Patterns (CSP) [10,11], Power Spectral Density (PSD) analysis [12], Short-Time Fourier Transform (STFT) [13], Wavelet Packet Decomposition (WPD) [14], Autoregressive Model (AR) [15], etc. Among them, CSP is an effective spatial filtering and feature extraction method, which can enhance the distinction between two-class MI signals by capturing spatial patterns in EEG signals. So far, CSP has achieved significant success in the MI-BCI field, leading to various CSP variants, the most representative of which is the filter bank CSP (FBCSP) algorithm [16]. FBCSP involves frequency band selection using specific filters, integrating frequency domain information, and subsequently extracting spatial features. Canonical classification algorithms include Linear Discriminant Analysis (LDA), Logistic Regression (LR), and Support Vector Machine (SVM). However, traditional ML methods usually separate these two steps and rely on manually crafted features guided by human prior knowledge and experience.

In recent years, DL has shown remarkable performance in image recognition, natural language processing (NLP) and speech recognition [17–19], driving the rapid development of artificial intelligence (AI). Against this backdrop, DL techniques are rapidly penetrating into BCI research. DL integrates the two steps of feature extraction and pattern classification, and can automatically extract abstract and high-level features from raw EEG signals. This end-to-end learning approach enhances the system’s performance and generalization ability, enabling it to better adapt to variations in subjects and tasks. Schirrmeister et al. [20] investigated deep convolutional neural networks (CNNs) with different architectures for decoding MI tasks from raw EEG signals. The study suggests that the CNN-based models achieved at least as good performance as the widely used FBCSP algorithm. Lawhern et al. [21] designed a compact and lightweight CNN, termed EEGNet, and applied it for four BCI paradigms, i.e., P300, error-related negative (ERN), movement-related cortical potential (MRCP), and sensorimotor rhythm (SMR). Wang et al. [22] introduced an EEG_GENet network, incorporating a feature-level graph embedding approach and combining it with the EEGNet. Following validation on two BCI datasets suggests that the method outperforms existing approaches compared to data-level graph embedding methods. Wu et al. [23] proposed a parallel multi-scale filter bank CNN (MSFBCNN) for MI decoding. They trained an end-to-end network to comprehensively extract the spatio-temporal features of EEG signals. Furthermore, they embedded the network structure into the EEGNet to validate the effectiveness of the proposed architecture. Chen et al. [24] designed a multi-branch network structure called FB-Sinc-SCANet, incorporating a filter bank with Sinc-convolution layers. Additionally, they integrated channel self-attention for feature selection (CSA-FS) from four frequency bands.

Recently, attention mechanism has attracted widespread attention and has been utilized in the field of BCI. The Transformer model [25] aims to address challenges faced by traditional DL models such as recurrent neural networks (RNNs) and long short-term memory (LSTM) networks when dealing with long sequential data. Wairagkar et al. [26,27] showed that time series such as EEG signals have long-term dependencies that can be characterized by long-range temporal correlation. Song et al. [28] introduced a compact EEG-Conformer network to capture long-term dependencies in MI-EEG signals, encapsulating both local and global features within a unified framework for EEG signal classification. Wang et al. [29] proposed a novel algorithmic structure combining EEG channel (i.e., spatial) attention with an Swin Transformer. This approach effectively leverages internal connections to narrow the gap between channels and targets, capable of capturing time-frequency-spatial features hidden in EEG data. Wang et al. [30] proposed an end-to-end DL model named SMANet, which effectively integrates a SincNet, a multibranch spatial-temporal CNN and a channel attention mechanism for MI-EEG decoding. It achieved superior accuracies over six state-of-the-art methods on a four-class MI dataset and two two-class MI datasets. In summary, incorporating temporal and channel attention into convolutional networks has attracted a lot of interests, showing great potential in performance improvement [24,26–33].

Although the above-mentioned DL models achieved better performance than traditional ML models, there is still space to promote the classification accuracy for building a robust and reliable BCI system. The performance of a DL model depends largely on its structure and the size of training data. For a given dataset, its size can be augmented to a certain extent using some method and the main challenge of applying DL in an MI-BCI consists in creating a suitable classification model. To address the problem, we propose an end-to-end DL network based on multi-scale CNNs and temporal and channel attention mechanisms (MSTCANet) for decoding MI-EEG data. The EEG data are first fed into four parallel DL branches, each of which includes sequentially connected temporal convolution, spatial convolution, and 2-dimentional average pooling layer, followed by a Transformer-based temporal attention block. Outputs from the four branches are concatenated along channel dimension and then an efficient channel attention network (ECANet) is applied to capture the dependency between feature channels. Finally, a CNN is used to predict the label of a single-trial EEG signal for reducing the computational complexity. The main contributions of this paper are as follows.

1) By efficiently incorporating multiscale spatial-temporal convolutions and the Transformer-based attention mechanism, the proposed network not only extracts comprehensive feature information, but also encapsulates both local and global features within a unified framework for MI-EEG signal classification, enabling the model to learn the global correlation of local temporal features.
2) By concatenating the feature signals from four branches and applying an efficient channel attention network (ECANet), the proposed network is capable of achieving the effective fusion of channel features via automatically adjusting weights to focus more on channels containing key information, significantly enhances the representation capability of feature signals.
3) Based on three public MI-BCI datasets comprising a total of 32 participants and four MI tasks, the performance of the proposed network is analyzed thoroughly in terms of decoding accuracy and standard deviation in inter-session scenarios, and compared with that of five state-of-the art (SOTA) networks. Experimental results validated the superiority of the proposed model.

## 2. Method

In this section, we first provide an overview of the proposed network, then detail each component of the network, and finally delineate its training strategy.

### 2.1. Overview

The proposed DL network is illustrated in Fig 1, which is based on multi-scale filtering and attention mechanism for extracting spatiotemporal features in EEG signals. The network consists of four modules, i.e., a multi-scale CNN module, a temporal self-attention module, a channel attention module and a classification module. They aim at extracting spatiotemporal features in multiple scales, capturing long-range temporal dependencies in EEG signals, fusing the feature maps from convolutional channels and classifying the fused feature signals into task labels, respectively.

**Fig 1:**
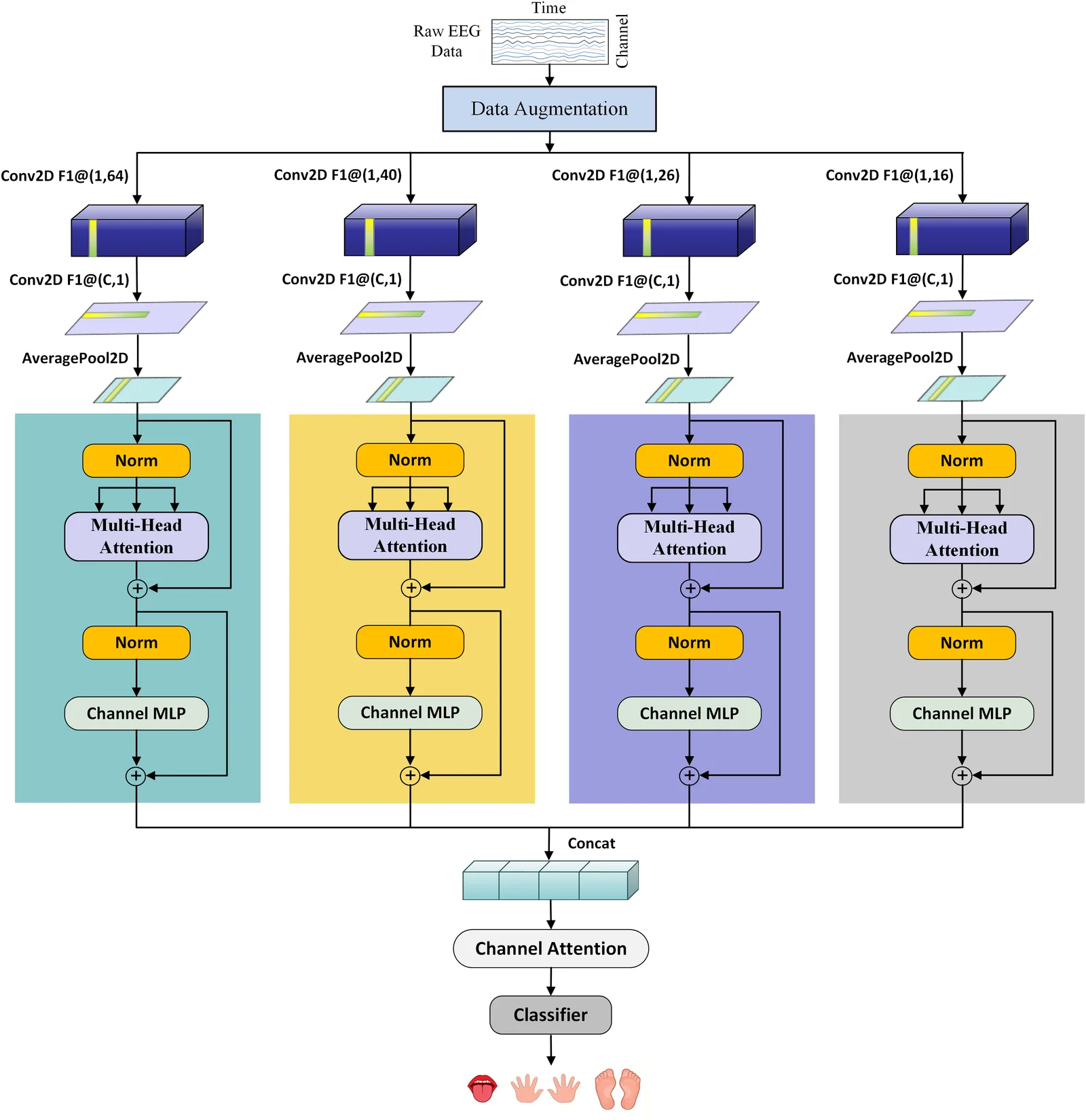
Structure of the proposed network. F1 and C denote the number of kernels and the number of EEG channels respectively.

### 2.2. Multi-Scale CNN module

The augmented EEG data are fed into the multi-scale CNN module consisting of four parallel CNN units, each of which comprises a temporal convolutional layer, a spatial convolutional layer and an average pooling layer. The temporal convolutional layers have varying kernel sizes, allowing the extraction of temporal features at multiple different scales. The spatial convolutional layers share the same kernel size for capturing spatial dependencies and topological structures among different EEG electrodes. The average pooling layers are used to reduce the dimensionality of feature signals and computational complexity. The parameters of the multi-scale CNN module are listed in Table 1.

**Table 1:** Parameters of the multi-scale parallel CNN module. tConv1 ~ 4 denote the four temporal convolutional layers, sConv1 ~ 4 represent the four spatial convolutional layers, and aPool1 ~ 4 denote the four average pooling layers. C and T are the numbers of EEG channels and sampling time points, respectively.

Layer	tConv1	tConv2	tConv3	tConv4	sConv1 ~ sConv4	aPool1 ~ aPool4
Input	(1, C, T)	(1, C, T)	(1, C, T)	(1, C, T)	C11 ~ C14	C21 ~ C24
Kernel	(1, 64)	(1, 40)	(1, 26)	(1, 16)	(C, 1)	(1, 75)
Stride	(0, 32)	(0, 20)	(0, 13)	(0, 8)	(1, 1)	(1, 15)
Padding	Same	Same	Same	Same	Same	–
# kernels (k)	40	40	40	40	40	40
Output	C11: (40, C, T)	C12: (40, C, T)	C13: (40, C, T)	C14: (40, C, T)	C21: (40, 1, T) C22: (40, 1, T) C23: (40, 1, T) C24: (40, 1, T)	C31: (40, 1, T1) C32: (40, 1, T1) C33: (40, 1, T1) C34: (40, 1, T1)

During the temporal convolution process, the convolution kernel of different scales (or filter sizes) extracts temporal feature maps corresponding to different frequency bands. Larger-size kernels capture coarse-grain information, whereas smaller-size kernels can retain fine-grain information [34,35]. Thereby, a single-scale convolutional network may be difficult to extract sufficient information for discriminating MI tasks. A CNN module with multi-scale kernel sizes is an effective strategy to preserve both low- and high-frequency information of EEG signals to improve the performance of MI decoding. Based on this idea, most of previous studies [31,36,37] created a multi-scale CNN based on filter bank techniques to extract features from multiple fixed frequency sub-bands such as δ (1–4 Hz), θ (4–8 Hz), α (8–13 Hz) and β (13–30 Hz). However, these frequency bands are prone to change for different subjects because of inter-subject variability and the non-stationary nature of EEG signals, and thus multi-scale CNNs with sub-band filtered input signals is not a good choice. Accordingly, a multi-scale CNN module that uses raw EEG signals as common input is adopted to simultaneously process information from different frequency bands. The input signal is of the dimension (C, T), where C represents the number of EEG channels, and T denotes the length of EEG signals (i.e., the number of sampling points).

The kernel sizes of the temporal convolutional layers are determined based on the characteristics of EEG signals and the sampling rate [26], and are set to 64, 40, 26, and 16 respectively. The maximum and minimum kernels (64 and 16) were determined as approximate fs/4 and fs/16 (fs is the sampling rate), then the remaining two kernels were selected between 64 and 16, and were ultimately determined experimentally to be 40 and 26 respectively. The four kernel sizes correspond approximately to the temporal windows of 250ms, 160ms, 100ms and 64ms respectively, and to the frequencies of 4 Hz, 6 Hz, 10 Hz and 16 Hz respectively. Thereby, the four kernels can be used to extract feature signals above 4 Hz, 6 Hz, 10 Hz and 16 Hz respectively. To maintain the same input and output sizes for subsequent concatenation, padding is set to half the size of the convolutional kernel. Regarding the spatial convolutional layers, the same convolutional kernel with size (C, 1) and stride (1, 1) is utilized to serve as spatial filters. The batch normalization is followed to maintain data stability. The average pooling layers are used to compress the temporal information of the spatially filtered signals and reduce the number of trainable parameters. Their kernel size is set to (1, 75) with a stride of (1, 15). After the multi-scale CNN module, the outputs of the four CNN branches are identical in size, facilitating subsequent feature fusion by concatenating them in channel dimension.

### 2.3. Temporal self-attention module

CNNs may only extract local temporal features, potentially failing to capture long-term dependencies in EEG signals. To address this limitation, we introduce the temporal self-attention module to learn the global temporal correlations of EEG signal features, thereby enlarging the limited receptive field of the single-scale CNN branches. The temporal self-attention module consists of four Transformer blocks, each of which follows a CNN branch.

Attention-based Transformer has become a significant milestone in NLP and other sequence modeling tasks [25]. It has demonstrated outstanding performance in various tasks. Its innovative structure has garnered widespread interest in the scientific community. By appending a Transformer block after a CNN branch, both local and global features are encapsulated within a unified EEG signal classification framework, facilitating learning global correlations in local temporal features.

Fig 2(a) shows the architecture of the Transformer block, which has two subblocks: the first one includes a normalization layer and a multi-head attention layer, whereas the second includes a normalization layer and a position-wise fully connected feed-forward layer. The residual connection is used around each of the two subblocks. As shown in Table 1, the output feature map of a CNN unit has the dimension (40, 1, T1), which is arranged as the feature map F with the dimension (T1, 40) by first squeezing the EEG channel dimension and then applying a transpose operation. All convolutional channels at each point in the temporal dimension are treated as a token and are fed into a Transformer block, where the self-attention mechanism is used to learn global temporal dependencies of EEG features, complementing the limited receptive field in a CNN unit. In addition, the multi-head strategy illustrated in Fig 2 (b) is employed in the Transformer block, aiming to further improve representation diversity. Subsequently, the tokens are equally divided into h segments (i.e., h heads) each with the dimension (T1, 40/h), which are fed into the Transformer block separately. The Transformer begins by transforming the feature map of a single head using a linear layer to obtain Query (Q), Key (K) and Value (V), formulated as


$$\begin{array}{c} Q_{\text{i}}=W_{i}^{q}\cdot LN\left(F_{i}\right),K_{\text{i}}=W_{i}^{k}\cdot LN\left(F_{i}\right),V_{\text{i}}=W_{i}^{v}\cdot LN\left(F_{i}\right) \end{array}$$ (1)

where i=1,2,···,h denotes the index of heads, $W^{q}$, $W^{k}$ and $W^{v}$ are the projection matrices of Q, K and V respectively, $F_{i}\in R^{T_{\text{1}}\times 40/h}$ denotes the divided feature map (i.e., the feature map of *i*th head), and LN signifies layer normalization.

**Fig 2:**
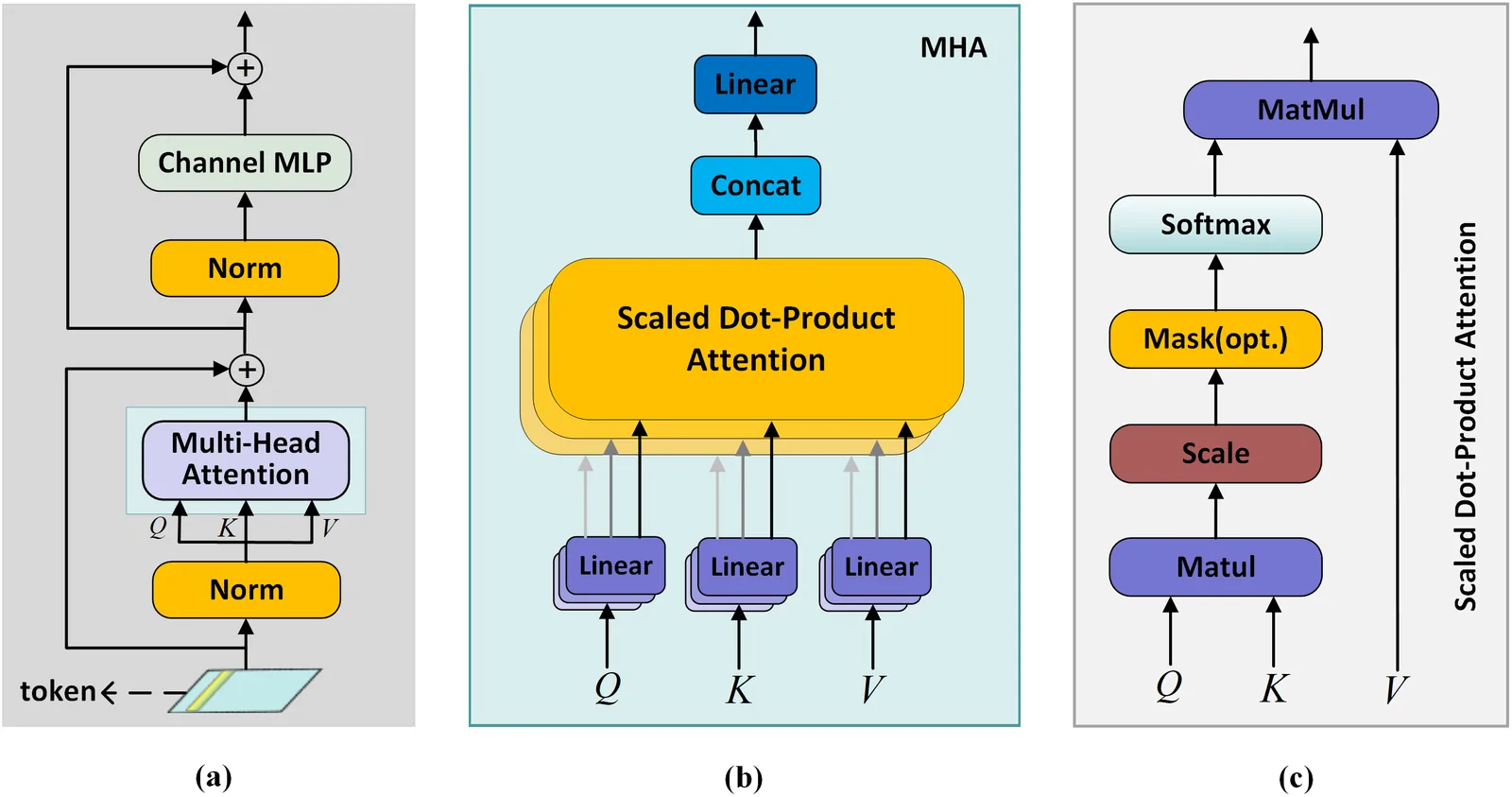
Transformer block: (a) Structure; (b) Multi-head attention; (c) Scaled dot-product attention.

Aiming to evaluate the correlation between different tokens, the scaled dot-product attention (SDPA) shown in Fig 2 (c) is used for performing the multi-head attention (MHA). For a single head i, SDPA calculates the dot product of all $K_{i}$ with $Q_{i}$, scaling each dot product by a factor of $\sqrt{d_{k}}$, and applying the Softmax function to obtain the weights for the $V_{i}$, as illustrated in Eq. (2). $d_{k}$ is the length of a token for a single head. The scaling factor $\sqrt{d_{k}}$ is used to mitigate the issue of gradient vanishing.


$$\begin{array}{c} Attention\left(Q_{i},K_{i},V_{i}\right)=softmax\left(\frac{Q_{i}K_{i}^{T}}{\sqrt{d_{k}}}\right)V_{i} \end{array}$$ (2)

In each Transformer block, the number of heads is set to h=10, and the outputs generated by all heads are concatenated together to form the final output:


$$\begin{array}{c} MHA\left(Q,K,V\right)=concat\left(head_{\text{1}},\cdots ,head_{h}\right)W^{o} \end{array}$$ (3)

where $head_{i}=attention\left(Q_{i},K_{i},V_{i}\right)$, and $W^{o}$ is the weight matrix for combining the output from multiple heads. By employing residual connections, the input features F are added to the output features of the MHA mechanism. This implies that the final output not only contains information from the MHA mechanism, but also retains the original input features. This residual connection aids in ensuring smooth gradient flow through the network, thereby enhancing training stability and effectiveness. The output of the Transformer block is formulated as follows:


$$\begin{array}{c} F_{o}=MHA\left(Q,K,V\right)+F \end{array}$$ (4)

The output of the Transformer block is passed through the Gaussian Error Linear Unit (GELU) non-linear activation function to generate the final output.

### 2.4. Channel attention module

The channel attention module is leveraged for feature channel fusion. Each of the four branch networks captures spatiotemporal features containing long-term temporal dependencies. In such scenarios, it is essential to adjust the weights of the features extracted by each branch network, allowing the network to concentrate more on the features crucial for the current MI task. We concatenate the output features of the four branches along the channel dimension and perform channel fusion using the ECANet [38], which only involves a handful of parameters while bringing clear performance gain.

The squeeze-and-excitation network (SENet) [39] is a commonly used channel attention mechanism aiming to enhance the network’s focus on specific channels by learning the importance of each channel. Although the SENet reduces model complexity by employing fully connected layers for dimensionality reduction, it disrupts the direct correspondence between channels and their weights. The ECANet addresses this issue by utilizing a one-dimensional convolution to capture global contextual information and adaptively adjust the weights across the entire channels. The overall structure of the algorithm is illustrated in Fig 3.

**Fig 3:**
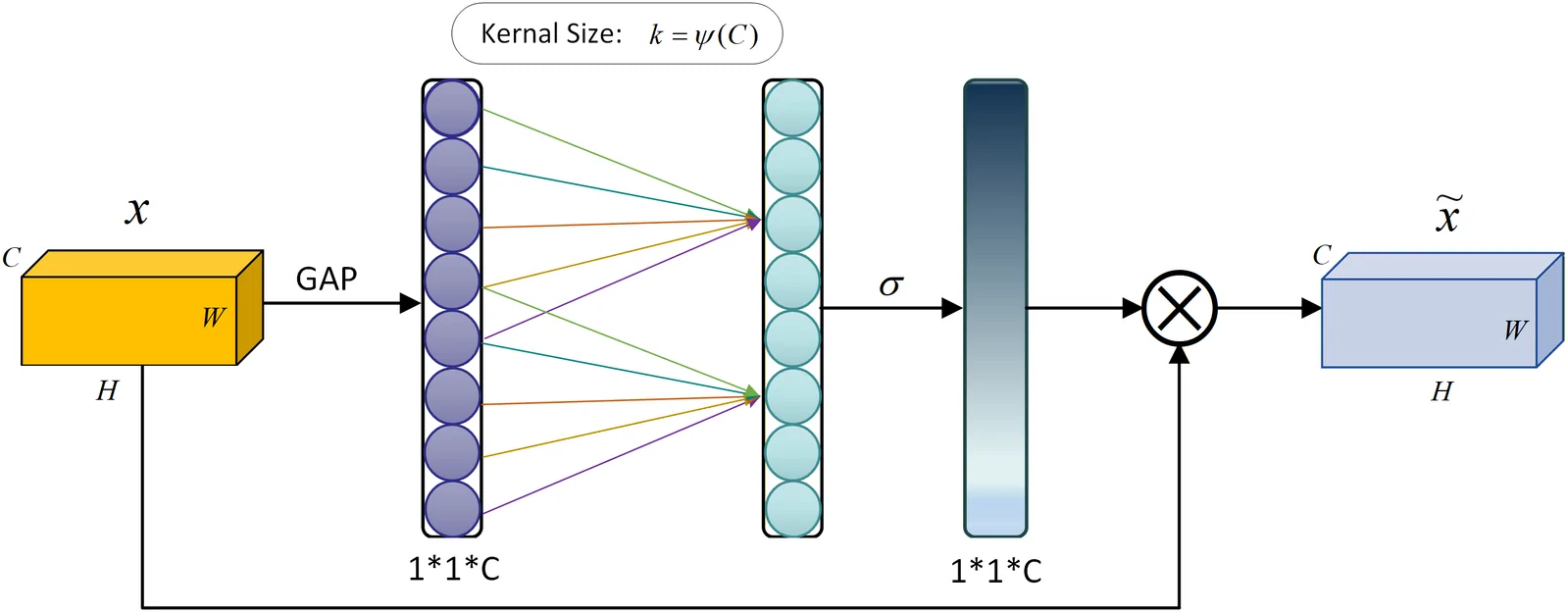
The network architecture of ECA mechanism.

Assume that the input feature map x has a size of (N, H, W, C), where N is the batch size. The ECANet starts by squeezing the feature map through channel-wise global average pooling (GAP), resulting in an output of (N, 1, 1, C). This step captures global information from the entire feature map. Subsequently, a one-dimensional convolution is applied to the pooled feature map (N, 1, 1, C) to calculate the channel weights, with the convolutional kernel size k defined as follows


$$\begin{array}{c} k=\psi \left(C\right)={\left|\left[{log}_{\text{2}}\left(C\right)+b\right]/\gamma \right|}_{odd} \end{array}$$ (5)

where $|t{|}_{odd}$ represents the closest odd integer to *t*, γ and b are two parameters tha*t* are set to 2 and 1 respectively. The channel weights are further mapped to the range of 0–1 through a Sigmoid function σ. Finally, the channels of an input feature map are weighted according to their respective weights. Through the non-linear mapping ψ, higher-dimensional channels have longer range interaction, whereas lower-dimensional ones undergo shorter-range interaction. The output of the channel attention module $\tilde{x}$ has the same size as its input x.

### 2.5. Classification module

The classification module serves as the final layer of the proposed network, responsible for transforming the feature representations output by the channel attention module into the ultimate class prediction results. Instead of using a fully connected layer and a Softmax function for predicting class labels, our classification module exploits a convolutional layer for classification, which has the kernel size of (71, 1) and the kernel count of $N_{c}$ (i.e., the number of MI classes). Compared to fully connected layers, convolutions primarily address weight sharing by removing redundant weight parameters, significantly reducing the number of weight parameters.

### 2.6. Model training

The proposed network is implemented using the PyTorch library in Python 3.10, utilizing the hardware configuration comprising an Intel Core i5-12400F CPU and an NVIDIA GeForce RTX 3080 GPU. The Glorot initializer is adopted to initialize all weights and the biases are set to zeros. The initial learning rate was set to 0.001. The dropout rate is set as 0.5 for the convolutional layers in multi-scale CNN module and 0.3 for the classification layer. The batch size per epoch is set as 160. The model is optimized with the joint loss function of cross-entropy loss and center loss. The cross-entropy loss is defined as


$$\begin{array}{c} L_{CE}=-\sum_{m=1}^{N_{C}}y_{m}log{\hat{y}}_{m} \end{array}$$ (6)

where $y_{m}$ and ${\hat{y}}_{m}$ are the true and predicted label respectively, and $N_{c}$ denotes the number of classes. The center loss aims to make all samples of the same class as close as possible to their class center in the feature space. It is formulated as follows


$$\begin{array}{c} L_{C}=\frac{\text{1}}{\text{2}}{\sum_{i=1}^{B}\|f_{i}-c_{yi}\|}_{\text{2}}^{\text{2}} \end{array}$$ (7)

where B, $f_{i}$ and $c_{yi}$ denote the batch size, the feature vector of the *i*th trial, and the feature center of category y to which the *i*th trial belongs. The total loss for model training is the weighted sum of cross-entropy loss and center loss and is formulated as follows


$$\begin{array}{c} L_{\text{total}}=L_{CE}+\lambda L_{C} \end{array}$$ (8)

where λ is the regularization parameter for adjusting the weight of the center loss within the total loss.

The augmented training data were divided in the ratio of 4:1 into a training set and a validation set. A two-stage training strategy was exploited to train the proposed model. In the first stage, the model was trained using only the training set, with the accuracy on the validation set monitored. The training phase ended when the number of training epochs reached 500 or when the validation accuracy stopped to increase within 100 consecutive training epochs. The model parameters that achieved the highest accuracy were saved; In the second stage, the model training started with these saved parameters using the whole training data, with the validation accuracy monitored. To prevent non-convergence, the training stage ceased when the number of training epochs reached 200.

## 3. Experimental data

In this study, three publicly available BCI datasets were used for evaluating the performance of the proposed model. The first two are dataset 2a and 2b from BCI Competition IV [40], and the third one is high gamma dataset (HGA) [41]. The main differences among the three datasets are that they have different numbers of subjects, trials, electrode channels and mental tasks. Access to the three datasets is granted upon agreeing to the terms and conditions outlined in the “Download of data sets” section. We followed the same procedure to obtain access for this study. In the original data acquisition, all subjects provided written informed consent for the use of their data in research under the assurance of confidentiality. In addition, these datasets were fully anonymized by the original data providers before being made accessible, ensuring no identifiable information was included.

### 3.1. Data acquisition

1) **Dataset 1:** The dataset contains MI-EEG data from 9 healthy participants, each of whom performed 2 sessions of experiments. Each session consisted of 288 trials, involving the four MI tasks of left-hand, right-hand, foot, and tongue, with 72 trials per task. EEG signals were recorded with 22 electrode channels covering the motor cortex at a sampling rate of 250 Hz. The first-session data were employed for model training and the second-session data for model testing.
2) **Dataset 2:** The dataset consists of MI-EEG data also from 9 healthy participants, each of whom performed five sessions of experiments. In contrast to dataset 1, dataset 2 involves the two MI tasks of left-hand and right-hand, collected through the three EEG channels C3, C4, and Cz, with a sampling rate of 250 Hz. Each of the first two sessions comprised 120 trials, 60 trials per task, whereas each of the last three sessions comprised 160 trials, 80 trials per task. The data from first three sessions were used for model training, and the data from last two for model testing.
3) **Dataset 3:** The HGA dataset comprises EEG data from 14 healthy participants, each performed two sessions. The first session includes approximately 880 trials per subject and the second consists of approximately 160 trials per subject. Each trial lasted for 4s and the subjects performed four MI tasks, i.e., left hand, right hand, feet and rest. EEG data were recorded at a sampling rate of 250 Hz on 128 electrodes, of which only 44 electrodes covering the motor cortex are used for the analysis. The first session was used for model training and the second for model testing.

### 3.2. Data preprocessing

For preprocessing these datasets, we employed the Braindecode framework [42], which is supported by PyTorch. The data segment of 4.5 s starting from 0.5s prior to the start of an MI task to the end of the task were intercepted for MI-EEG decoding. Thereby, a single trial has the dimension of (C, 1125), where C is the number of electrode channels. In addition, the z-score normalization is applied to these datasets, scaling the data to have a zero mean and unit standard deviation.

Considering the relatively small size of these datasets, the training sets were augmented using the approach segmentation and reconstruction (SR) [43]. Its idea is to first divide each training trial into several segments, and then generate new artificial trials as a concatenation of segments in chronological order, which come from different and randomly selected trials from the same class. In the study, the training trials of length 4.5s were divided into 9 segments, 0.5s per segment, and the number of generated artificial trials was 50 per task. This approach preserves the temporal correlation of the original data, aiding the model in learning their temporal information.

## 4. Results

Based on the aforementioned three MI datasets, we conducted classification experiments on the proposed MSTCANet model. Firstly, we compare our model with five SOTA models in terms of classification accuracy and standard deviation, and showcase the confusion matrices of the proposed model. Then, we conducted ablation experiments on the proposed model, progressively integrating modules and presenting dimensionality reduction data. Next, we exhibit the influence of different channel attention mechanisms on decoding performance. Finally, we analyze the effectiveness of data augmentation on the three MI datasets.

### 4.1. Comparison with SOTA methods

The proposed model was compared with the following five SOTA models: 1) EEGNet [21]; 2) Shallow ConvNet [20]; 3) CNN+LSTM [44]; 4) MSFBCNN [23]; 5) FBCNet [45]. Tables 2–4 report the classification results on datasets 1, 2 and 3 respectively. From these tables, it can be observed that the MSTCANet model achieved the highest average accuracies of 80.21%, 90.19% and 97.32% on datasets 1, 2 and 3 respectively, surpassing the strongest baselines FBCNet on Dataset 1, MSFBCNN on Dataset 2 and MSFBCNN on Dataset 3 by 2.59%, 1.27%, and 1.99% respectively. For datasets 2 and 3, the standard deviations yielded by MSTCANet are much lower than those yielded by other models; For dataset 1, the standard deviation yielded by MSTCANet shows no significant difference compared to those yielded by the five baseline models except for CNN+LSTM. This means that the proposed network not only has the best classification performance, but also has classification robustness. Paired t-tests were conducted between the proposed model and each of five baseline models. The results show that for dataset 1, the proposed model significantly outperforms EEGNet, Shallow ConvNet, MSFBCNN, CNN+LSTM, and FBCNet with *p* values (effect sizes denoted by Cohen’s d) equaling 0.0488 (0.7738), 0.0252 (0.9155), 0.0495 (0.2930), 0.0491(0.4341) and 0.0130 (1.0597) respectively; For dataset 2 and 3, the proposed model significantly outperforms the five baseline models with all p values less than 0.05.

**Table 2:** Classification accuracy of the six compared models achieved by each of the nine subjects in dataset 1 and their average accuracy (Mean±Std, %). The best results are highlighted in bold.

Methods	Subject									Mean	Std
	A01	A02	A03	A04	A05	A06	A07	A08	A09		
EEGNet	86.11	61.81	91.32	62.50	68.06	59.03	88.19	82.99	**88.54**	76.51	13.34
Shallow ConvNet	84.38	62.15	90.28	73.96	70.49	56.94	91.67	86.11	81.94	76.48	12.38
MSFBCNN	84.03	61.11	**93.40**	**76.39**	73.61	60.07	82.29	86.11	80.90	77.55	12.23
CNN+LSTM	81.25	**64.93**	89.58	71.18	**74.31**	**67.01**	77.43	85.07	84.72	77.28	**8.57**
FBCNet	85.07	58.68	93.06	73.26	71.18	62.50	88.89	81.25	84.72	77.62	11.90
MSTCANet	**89.58**	60.07	92.01	**76.39**	70.83	64.58	**94.44**	**87.15**	86.81	**80.21**	12.62

**Table 3:** Classification accuracy (%) of the six compared models achieved by each of the nine subjects in dataset 2 and their average accuracy (Mean±Std, %). The best results are highlighted in bold.

Method	Subject									Mean	Std
	B01	B02	B03	B04	B05	B06	B07	B08	B09		
EEGNet	77.19	65.36	84.06	95.31	93.75	82.50	87.19	92.19	85.31	84.76	9.31
Shallow ConvNet	78.75	71.07	85.00	96.56	97.81	87.19	**93.44**	**94.69**	89.06	88.17	8.85
MSFBCNN	78.44	71.88	85.00	98.13	97.81	91.19	88.13	94.06	**95.63**	88.92	9.06
CNN+LSTM	78.13	72.81	83.75	98.44	95.31	90.31	81.56	94.06	92.19	87.40	8.71
FBCNet	75.00	71.56	81.56	97.81	93.75	90.63	82.19	92.19	89.69	86.04	8.92
MSTCANet	**81.56**	**73.57**	**85.63**	**99.06**	**99.69**	**91.25**	93.13	**94.69**	93.13	**90.19**	**8.51**

**Table 4:** Classification accuracy (%) of the six compared models achieved by each of the nine subjects in dataset 3 and their average accuracy (Mean±Std, %). The best results are highlighted in bold.

Subject	Method					
	EEGNet	Shallow ConvNet	MSFBCNN	CNN+LSTM	FBCNet	MSTCANet
S01	**96.88**	95.62	95.50	94.38	93.13	95.63
S02	95.63	97.50	96.88	92.50	93.75	**97.50**
S03	99.38	99.38	**100**	95.63	99.38	98.75
S04	**99.38**	97.50	98.75	97.50	98.75	**99.38**
S05	96.26	98.13	96.88	93.75	96.88	**100**
S06	92.50	**98.13**	**98.13**	90.00	97.50	**98.13**
S07	94.34	94.34	91.19	91.19	87.42	**94.97**
S08	93.13	98.13	95.00	93.13	**99.38**	**99.38**
S09	**100**	93.12	96.88	91.88	88.13	99.38
S10	**96.25**	91.88	92.50	95.63	95.63	**96.25**
S11	83.75	98.75	97.50	83.75	84.38	**99.38**
S12	97.5	96.88	98.12	96.25	97.50	**99.38**
S13	94.97	96.23	96.86	94.97	96.86	**98.74**
S14	80.63	79.38	83.75	71.88	80.00	**85.63**
Mean	94.33	95.36	95.53	91.60	93.48	**97.32**
Std	5.64	5.09	4.24	6.62	6.12	**3.71**

### 4.2. Confusion matrix

The confusion matrix visualizes the classification accuracy of the proposed MSTCANet for each category, including the counts of true positives, true negatives, false positives and false negatives, and thereby provides a more intuitive depiction of the model’s classification outcomes. Fig 4 illustrates the averaged confusion matrix of the testing data across all subjects in each of three datasets. As shown in the figure, the horizontal axis represents the model’s predicted category labels, whereas the vertical axis denotes the true category labels. Each cell in a confusion matrix indicates the count of trials where the predicted category intersects with the true category. For example, the element in the *i*th row and *j*th column represents the count of samples where the model predicted category *j* while the true category was *i*. For the dataset 1, the most easily confused task is MI of Tongue, whereas the other three tasks have essentially the same degree of confusion; For the dataset 2, the MI of right hand is more easily confused than that of left hand; For the dataset 3, there are small differences in the degree of confusion among the four MI tasks.

**Fig 4:**
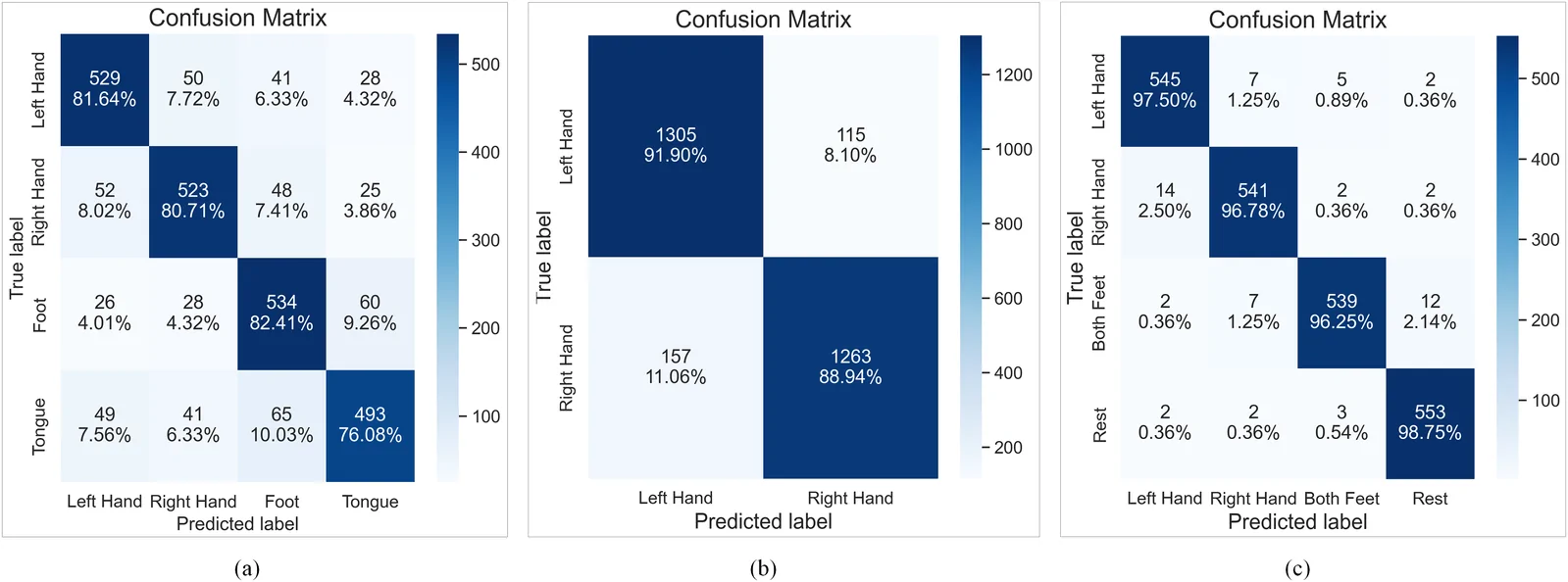
The averaged confusion matrix of testing trials across all subjects in (a) dataset 1, (b) dataset 2, and (c) dataset 3, yielded by the proposed model MSTCANet. Each cell in a confusion matrix indicates the count of trials where the predicted category intersects with the true category.

### 4.3. Ablation study

To validate the effectiveness of key modules in the proposed model for feature processing, we conducted ablation experiments on the model across three datasets. This study investigated three models: 1) Model 1: the degenerate model including only the multi-scale CNN module and the classification module; 2) Model 2: the degenerate model by adding the temporal self-attention module to Model 1; 3) Model 3: the degenerate model by adding the channel attention module to Model 2, i.e., the complete MSTCANet model. The feature distribution derived from unseen testing data are visualized using the dimensionality reduction technique t-SNE [46]. The averaged accuracy across all subjects in each dataset serves as a quantitative metric.

Taking a typical subject in every dataset as an example, Figs 5–7 illustrate the feature distributions yielded by the three models at the classification layer, with each color denoting a different class. As a contrast, the distributions of raw testing data are also displayed in the three figures. It is revealed from the figures that the original data distributions of the four categories (Fig 5(a) and Fig 7(a)) or two categories (Fig 6(a)) are merged and fail to be separated at all; The features extracted by Model 1 can basically be divided into four clusters (Fig 5(b) and Fig 7(b)) or two clusters (Fig 6(b)), but the distances among them are very small and the boundaries are quite blurred, resulting in inaccurate decoding results. Thereby, the multi-scale CNN module demonstrates efficacy in the feature extraction of EEG signals and establishes a solid foundation for decoding MI tasks; The boundaries among the four clusters (Fig 5(c) and Fig 7(c)) or two clusters (Fig 6(c)) yielded by Model 2 are more distinct and increasingly discernible, explaining that embedding the temporal self-attention module in Model 1 can effectively raise the decoding accuracy; The feature clustering per task yielded by Model 3 (i.e., the MSTCANet model) is strengthened further, and their boundaries are more apparent, suggesting that incorporating the ECANet module in Model 2 can enhance the feature representation capability of EEG signals and the consequent decoding performance.

**Fig 5:**
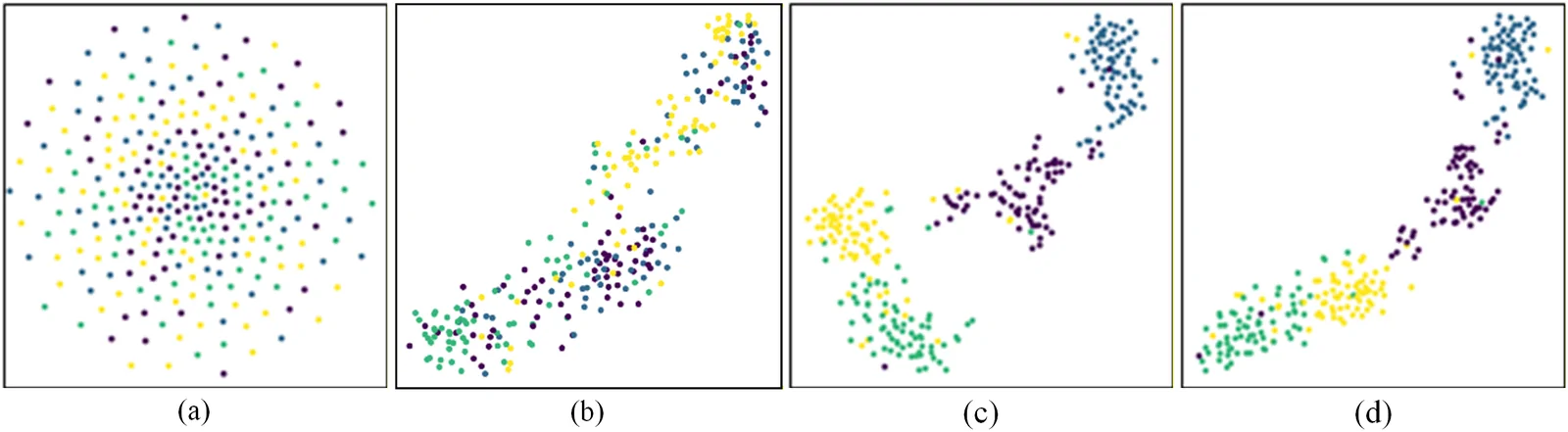
The scatter plots of original data (a) and the features extracted by Model 1 (b), Model 2 (c) and Model 3 (d) for the subject A03 in dataset 1. Each dot represents a testing trial. The visualization approach t-SNE is used to transform high-dimensional data to two dimensions. Yellow, green, blue and purple colors denote MI of left hand, right hand, foot and tongue respectively.

**Fig 6:**
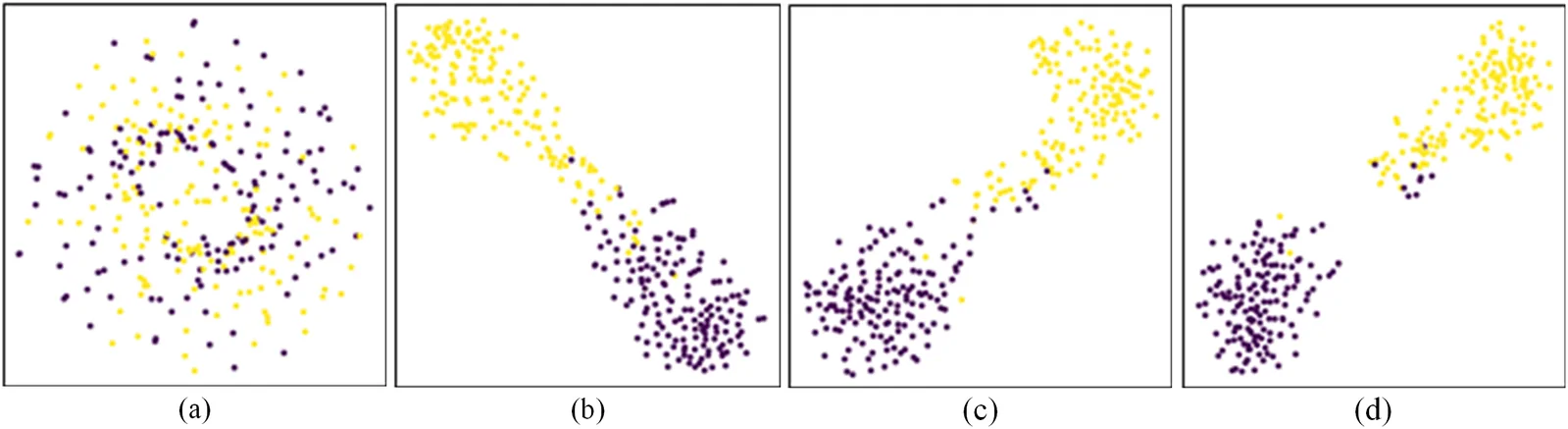
The scatter plots of original data (a) and the features extracted by Model 1 (b), Model 2 (c) and Model 3 (d) for the subject B04 in dataset 2. Each dot represents a testing trial. The visualization approach t-SNE is used to transform high-dimensional data to two dimensions. Yellow and purple colors denote MI of left hand and right hand respectively.

**Fig 7:**
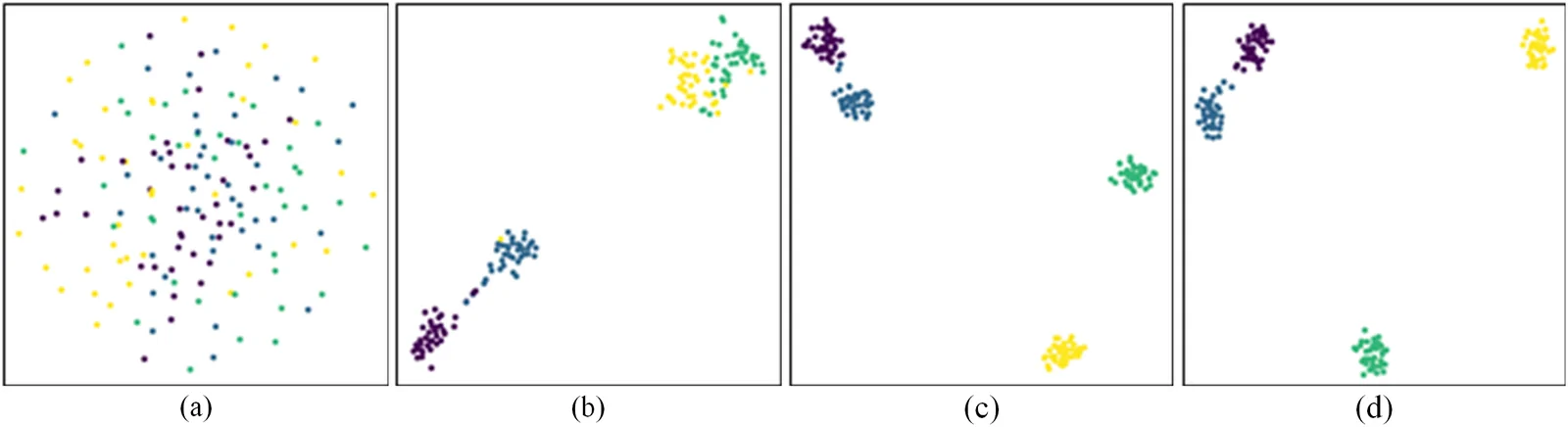
The scatter plots of original data (a) and the features extracted by Model 1 (b), Model 2 (c) and Model 3 (d) for the subject S05 in dataset 3. Each dot represents a testing trial. The visualization approach t-SNE is used to transform high-dimensional data to two dimensions. Yellow, green, blue and purple colors denote MI of left hand, right hand, foot and rest respectively.

Table 5 reports the averaged decoding accuracy of the three models across all subjects in each of the three datasets. It is revealed from the table that on the three datasets, Model 1 yields relatively high accuracies, Model 2 achieves higher accuracies than Model 1, and Model 3 (the proposed model) yielded the highest accuracies among the three models. Paired t-tests were performed between Model 3 and each of the other two models. The statistical analyses exhibit that Model 3 significantly outperforms the other two models with all p value less than 0.05.

**Table 5:** The averaged accuracy (Mean±Std, %) of the three models across all subjects in a dataset.

Data set	Model 1	Model 2	Model 3
Dataset 1	77.47 ± 13.33	78.43 ± 12.40	**80.21 ± 12.62**
Dataset 2	88.67 ± 8.25	89.48 ± 8.51	**90.19 ± 8.02**
Dataset 3	95.76 ± 4.22	96.83 ± 4.02	**97.32 ± 3.71**

These qualitative and quantitative analyses indicate that each of three key modules, the multi-scale CNN module, the temporal self-attention module and channel attention module, is conducive to improving the decoding performance of the proposed model.

### 4.4. Influence of channel attention mechanisms

Channel attention mechanism is a crucial approach to enhancing the feature representation of EEG signals. However, different channel attention mechanisms have significant differences in improving network performance. We explored the impacts of three channel attention mechanisms on the decoding accuracy of the proposed model. Fig 8 shows the averaged accuracies of CBAM [47], SENet [39] and ECANet [38] across all subjects in each of the three datasets. From the figure, it can be observed that ECANet yielded higher accuracy than CBAM and SENet on each dataset. Paired t-tests show that ECANet significantly outperforms CBAM with p < 0.01 and SENet with p < 0.05. Thereby, ECANet is exploited for channel attention in the proposed model.

**Fig 8:**
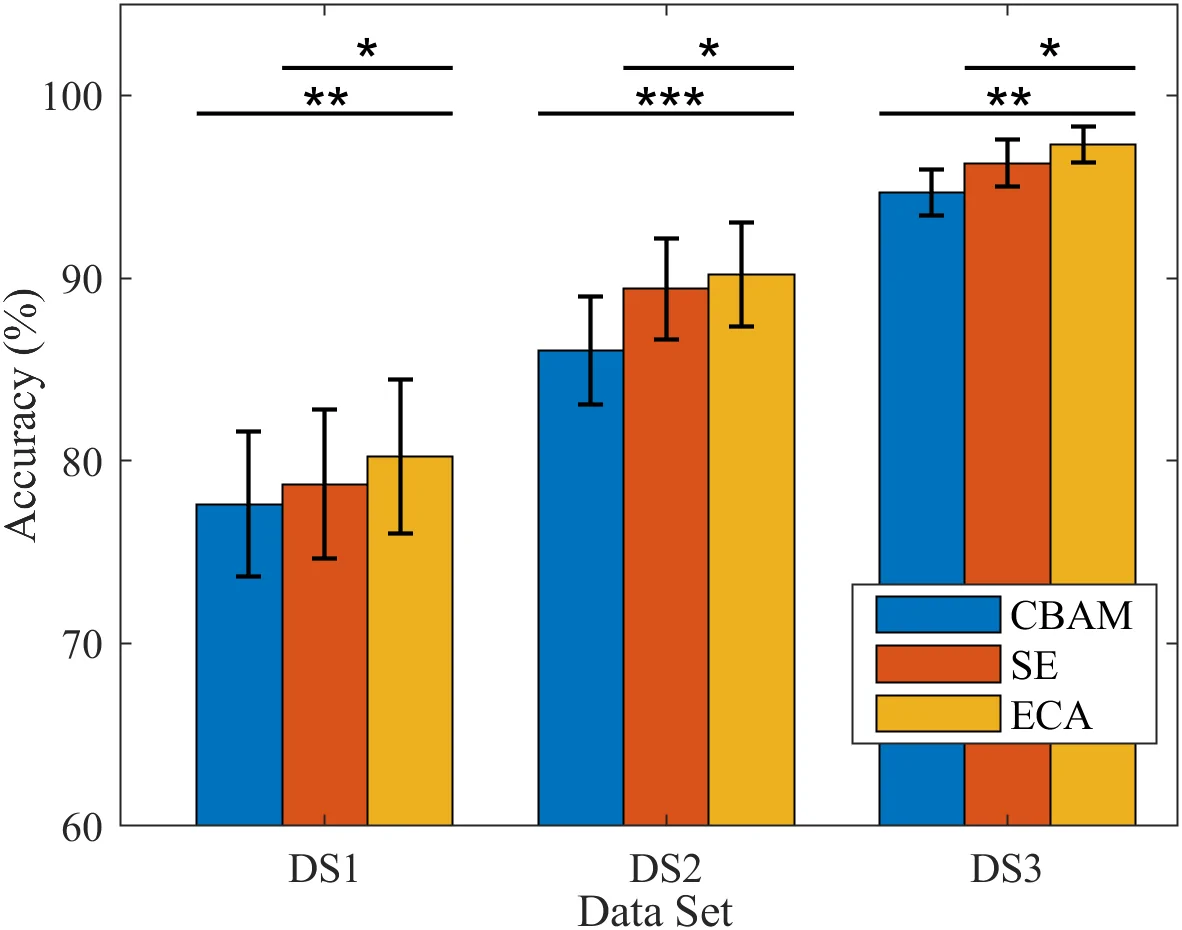
The averaged classification accuracies of the three channel attention mechanisms across all subjects in each of the three datasets. The error bars represent the standard error. The asterisks *, ** and *** denote p < 0.05, p < 0.01 and p < 0.001 respectively.

### 4.5. Effectiveness of data augmentation

The performance of a DL model depends not only on its network architecture but also on the volume of training data. Due to the insufficient number of training trials in the three MI-BCI datasets, the decoding performance of the proposed model is limited to some extent. Data augmentation is an effective method for improving the decoding accuracy and generalization ability of the proposed model. The SR strategy is adopted to augment the amount of training trials during each training epoch. Fig 9 depicts the classification accuracies of all subjects before and after data augmentation on each of the three datasets. It is revealed from the figure that for dataset 1 and 2, the accuracy of each subject improved after data augmentation; For dataset 3, after data augmentation, accuracies of nine subjects increased, accuracy of one subject (S05) remained unchanged, and accuracies of four subjects (S01, S03, S06, and S07) decreased. The reason why data augmentation was ineffective for the five subjects might be attributed to individual discrepancy among subjects in the dataset, which means that for the five subjects, this data augmentation approach did not increase the diversity of their data distribution and instead actually amplified noise interference, as well as to the inherent limitations of this augmentation approach, which operates solely in the time domain and is unable to effectively capture the dynamic changes of EEG signals in the frequency or spatial domains. On average, the proposed model with data augmentation outperformed that without data augmentation on all the three datasets. Paired t-tests suggest that the averaged accuracy across all subjects in a dataset after data augmentation is significantly higher than that without data augmentation on datasets 1 and 2 with *p* < 0.01, and on dataset 2 with p < 0.05. These results validate the effectiveness of data augmentation in general.

**Fig 9:**
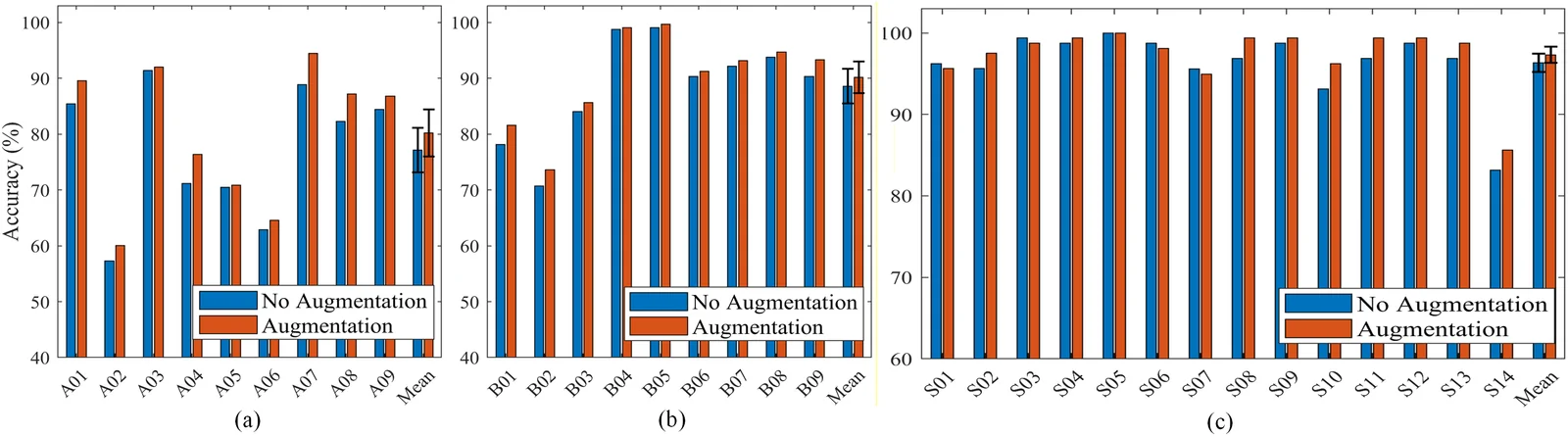
The classification accuracies of all subjects and their averages before and after data augmentation on (a) datasets 1, (b) dataset 2 and (c) dataset 3. The error bars denote the standard deviation.

### 4.6. Computational Complexity

Model complexity depends not only on the model’s structure but also on the training dataset. Taking the dataset BCIC-IV-2a as an example, the parameter count, floating-point operations (FLOPs), training time, testing time and decoding accuracy of each of the six compared models is listed in Table 6. The proposed model has the largest number of parameters among all the compared models, resulting in maximum Flops and training time. However, the number of FLOPs (627.174 K) is not a problem for modern computers, and the training time (2526 s) is not a serious problem as well because the model can be trained prior to a BCI experiment. Compared to the best baseline model MSFBCNN on the dataset, the increased training time resulted in a remarkable accuracy gain of 2.66%. In practical applications, the testing time is a more critical factor, as it determines whether a model can operate in real-time. The testing time for all the six compared models is very short and can fully meet the requirements of online applications.

**Table 6:** The computational complexity of MSTCANet and the five baseline models on the dataset 1.

Model	Parameter count (K)	FLOPs (M)	Training time (s)	Testing times (s)	Accuracy (%)
EEGNet	3.700	27.431	336	0.079	76.51
Shallow ConvNet	47.324	126.312	476	0.071	76.48
MSFBCNN	27.884	343.751	808	0.129	77.55
CNN+LSTM	81.068	133.811	346	0.095	77.28
FBCNet	8.356	2.592	436	0.084	76.20
MSTCANet	246.809	627.174	2562	0.131	80.21

### 4.7. Subject-independent classification

For the subject-independent classification, a leave-one subject-out strategy was used to split the overall dataset into a training set and a test set. Specifically, each subject in a dataset acted as testing subject and his/her data acted as testing set for classification, whereas the data from each of other subjects were put together as the training set to train the testing subject’s decoding model. Subsequently, the trained model was used to classify the testing data. Finally, the decoding accuracies of all subjects were averaged to acquire the total accuracy of the dataset. The subject-independent classification results are reported in Table 7. The table revealed that the decoding accuracies of MSTCANet are consistently higher than each baseline model on all the three datasets; Significant accuracy improvements exist between MSTCANet and all baselines on dataset 3 (p < 0.05) and between MSTCANet and all baselines except for MSFBCNN on dataset 1 and 2.

**Table 7:** Classification accuracies (Mean±Std, %) achieved by the proposed MSTCANet and the five baseline methods under the subject-independent scenario on the three MI-BCI datasets.

Dataset	Method					
	EEGNet	Shallow ConvNet	MSFBCNN	CNN+LSTM	FBCNet	MSTCANet
Dataset 1	56.85 ± 15.82	64.77 ± 3.01	67.24 ± 12.77	60.40 ± 8.30	46.37 ± 9.75	67.75 ± 13.70
Dataset 2	76.51 ± 5.32	75.45 ± 7.34	78.57 ± 8.16	77.25 ± 6.00	71.46 ± 9.54	79.61 ± 7.57
Dataset 3	58.31 ± 16.04	72.47 ± 12.18	75.47 ± 11.35	71.23 ± 10.87	59.86 ± 11.59	77.48 ± 10.28

## 5. Discussions

In this study, we propose a novel MSTCANet model, aimed at extracting richer and more robust features of MI-EEG signals and thereby improving the classification accuracy of MI-BCI systems. The proposed model effectively integrates three crucial modules: 1) a parallel multi-scale CNN module; 2) a Transformer-based temporal attention module; and 3) an ECANet-based channel attention module. The key innovative aspect of this model lies in that the parallel multi-scale network enables the simultaneous use of information from multiple scales and thereby comprehensively capturing features of EEG signals.

The multi-scale CNN module is capable of simultaneously processing information from different scales to address potential limitations of single-scale convolutions in the receptive field, laying a solid foundation for accurately decoding MI tasks. The temporal attention module is employed to learn the global temporal correlations of EEG signals, overcoming the CNN’s shortcoming of extracting only local temporal features. By appending a Transformer block after each convolutional unit, both local and global features are captured within a unified framework, enabling the learning of global correlations of local temporal features. The channel attention module adaptively adjusts the weights of channel features yielded by four DL branch networks, allowing the proposed model to focus more on features that are important for the current MI task. By intelligently integrating these three key modules, the proposed network captures robust and discriminative features, thereby possessing outstanding capability to decode EEG signals.

In the experiments of data analyses, the proposed model yielded the best results compared to five state-of-the-art DL models across three datasets, with average accuracy improvements of 2.59%, 1.27%, and 1.99% on datasets 1, 2 and 3 respectively. The confusion matrices were provided to explore which MI tasks are easily misclassified by the proposed model, indicating that the model achieved a good class balance in decoding MI tasks. The ablation study demonstrates that all the three key modules in the model play an effective role in boosting the model’s performance. The multi-scale CNN module yielded good classification accuracies across all three datasets, whereas both temporal and channel attention modules further enhance the decoding performance of the model. Feature visualization also demonstrated that the three modules are indispensable for classifying MI-EEG signals. Finally, we investigated whether data augmentation affects the model’s performance, verifying the efficacy of the SR approach in cases of scarce training data.

In recent years, DL technologies have seen significant advancement in the field of BCI research, greatly expanding their range of applications. Zhang’s research group conducted three outstanding studies [48–50] on spiking architectures, graph-based EEG modeling, and multi-domain attention designs. One study proposed a customed spiking neural network (SNN) for emotion recognition and MI decoding [48], which integrates an adaptive graph convolution and an LSTM block. The empirical evaluations on two public datasets demonstrate that SGLNet consistently outperforms existing state-of-the-art EEG classification algorithms, providing a new perspective for exploring high-performance SNNs for future BCIs. Another study presented a temporal-frequential attentional convolutional network (TFAC-Net) for driver drowsiness recognition using single-channel EEG [49]. The continuous wavelet transform is first used to generate a spectral-temporal representation, the temporal-frequential attention mechanism is then adopted to reveal critical time-frequency regions in terms of the driver’s mental state, and an adaptive feature fusion module is applied to integrate the most relevant feature channels for final prediction. The experimental results on a public EEG dataset demonstrate that the TFAC-Net is superior to the state-of-the-art methods, and could discover discriminative temporal-frequential regions. The third study proposed a two-stage domain adaptation with hybrid adaptive graph learning (TAHAG) strategy for emotion recognition [50], which involves aligning the refined node features across different domains via discrepancy alignment and capturing domain-invariant summarized features of the entire graph via adversarial training. Extensive experiments on three public datasets indicate the superiority of TAHAG compared to existing methods. Wang’s research group conducted three outstanding studies on spiking architectures, hybrid BCI research and graph-based EEG modeling [51–53]. The first study [51] proposed a spiking neural network-based classification method for decoding multiple MI tasks with a FBCSP algorithm to extract the spatio-temporal-frequency features of MI signals and the F-score to optimize and select these features. Experimental results on two public datasets showed that the average accuracy of the proposed framework reached up to 90.09% (kappa: 0.868) and 81.33% (kappa: 0.751) respectively. The second study [52] investigated a general decoding framework MetaNIRS for fNIRS-based motor execution/imagery, where a novel long-range dilation multilayer perceptron (LongDilMLP) is proposed to use the hemodynamic characteristics of fNIRS, and is integrated with the PoolFormer framework. The experimental results demonstrated that the average accuracies of 77.24%, 58.55%, and 85.52% on two public ME datasets and one self-collected MI dataset respectively. The third study [53] proposed a hybrid EEG-fNIRS decoding framework with dynamic graph convolutional-capsule networks for MI/ME tasks. A cascade structure of dynamic graph convolutional and capsule networks is used for accurate decoding of MI based BCIs with both EEG and fNIRS signals. A temporal convolution block, a dynamic graph convolution block, and a capsule generation block are used for learning temporal, spatial and capsuled features respectively. The capsuled features go through a cross-attention block, a feature fusion block and a dynamic routing block that is designed to learn the connection weights between primary capsules and digit capsules. The leave-one-session-out accuracies of 92.60% and 92.20% were achieved on a self-collected EEG-fNIRS dataset and a public dataset respectively, whereas the randomized five-fold cross-validation accuracy of 85.30% was yielded on another public dataset.

While the proposed model has superior decoding performance, several limitations exist in the study. Firstly, the study does not consider the influence of varying frequency bands on MI decoding. Recent studies [23,24] have shown that filtering MI signals via a filter bank or a Sinc convolution can provide distinct information contributing to task recognition. Thus, improving decoding performance by combining information from different sub-bands will be a research focus in the future. Secondly, the present study mainly explores the problem of decoding MI tasks in inter-session scenarios. The cross-subject classification [54] will be another research focus in the future. Transfer learning [55] may be an avenue to enhance the generalization ability of our model. Finally, the validity of the model was solely evaluated on MI paradigm, and future work will explore its application in other paradigms such as SSVEP and emotion recognition.

## 6. Conclusion

This paper proposes a new end-to-end network MSTCANet for MI-EEG classification. It first utilizes four parallel DL networks to extract multi-scale spatiotemporal features, acquiring more comprehensive feature information with different convolutional kernels. Then the Transformer temporal attention module is applied to capture long-term dependencies of temporal features, enhancing the representation capability of features. Subsequently, the output feature maps of the four Transformer blocks are fused through ECA-based channel attention module, enabling the model to focus more on channels containing key feature information. Data augmentation based on the SR strategy is exploited to raise the decoding performance by addressing the problem of insufficient training data. The proposed model is analyzed and evaluated on three MI-BCI datasets. Experimental results demonstrate that the model achieves superior decoding accuracies over five baseline models, confirming its potential in facilitating the applications of MI-BCI systems in real world.
